# Cucurbitacin: Ancient Compound Shedding New Light on Cancer Treatment

**DOI:** 10.1100/tsw.2010.44

**Published:** 2010-03-05

**Authors:** Dhong Hyun Lee, Gabriela B. Iwanski, Nils H. Thoennissen

**Affiliations:** ^1^ Division of Hematology and Oncology, Cedars-Sinai Medical Center, School of Medicine, University of California, Los Angeles, CA, USA; ^2^ Department of Biomedical Engineering, University of California, Los Angeles, CA, USA

**Keywords:** cucurbitacin, cancer, STAT3 inhibitor, JAK-STAT pathway, ERK inhibitor, MAPK pathway, synergism, chemotherapy, doxorubicin, gemcitabine

## Abstract

Cucurbitacins and their derivatives are triterpenoids found in medicinal plants known for their diverse pharmacological and biological activities, including anticancer effects, throughout human history. Although initial attention to cucurbitacin as a potential anticancer drug withered for decades, recent discoveries showing that cucurbitacin is a strong STAT3 (Signal Transducers and Activators of Transcription-3) inhibitor have reclaimed the attention of the drug industry one more time. There is increasing evidence showing that some cucurbitacins not only inhibit the JAK-STAT pathway, but also affect other signaling pathways, such as the MAPK pathway, which are also known to be important for cancer cell proliferation and survival. Moreover, some reports have shown the synergistic effect of cucurbitacins with known chemotherapeutic agents, such as doxorubicin and gemcitabine. In this review, we will summarize the recent discoveries regarding molecular mechanisms of action of cucurbitacins in human cancer cells and discuss the possibilities of cucurbitacin as a future anticancer drug in clinical settings.

## INTRODUCTION

A variety of plants used in native folk medicine around the world have been a good source of therapeutic agents. More than half of the commercially available drugs so far are plant derived or mimics of plant-derived substances[1,2,3]. Compared to chemically synthesized drugs, plant-derived drugs are natural products, and their efficacy and side effects have been empirically tested throughout human history. However, the lack of understanding of the molecular mechanism of the active compounds, as well as the presence of other chemicals in the plant extracts with unknown properties, have been the two biggest obstacles in the development of plant-based drugs. Therefore, the number of plant-derived drugs so far is much smaller than the number of medicinal plants known.

More problems emerge when it comes to the development of a plant-derived anticancer drug. Since many medicinal plants are "assumed" to have anticancer activity based on the description from the old medical records, it is imperative that they undergo intensive pharmacological and biological studies to prove their efficacy. For this reason, there are only a few plant-derived drugs currently available on the market for the treatment of human cancers. According to Cragg and Newman, there are four major groups of plant-derived anticancer drugs in clinical use[1]: vinca alkaloids (e.g., vinblastine and vincristine), epipodophyllotoxin derivatives (e.g., etoposide and teniposide), taxanes (e.g., paclitaxel [taxol]), and camptothecin derivatives (e.g., topotecan and irinotecan). However, the fact that there are still thousands of plant-derived compounds believed to have antitumor activity enhances the possibility for the discovery of new potent agents in cancer therapy.

In this review, we will discuss the possibility of one of the plant-derived compounds, cucurbitacin, as a potential anticancer drug. We will mainly focus our review on selected studies performed on human cancers using cucurbitacins and/or their derivatives from the 1990s. Recent advances in the understanding of the molecular mechanism of anticancer activity of cucurbitacin will also be discussed in detail.

## CUCURBITACIN, OLD DRUG WITH ANTICANCER ACTIVITY

Cucurbitacins refer to a group of tetracyclic triterpenoids initially identified in the plant family of *Cucurbitaceae*. In traditional medicine, cucurbitacin-containing plants have been known for their antipyretic, analgesic, anti-inflammatory, antimicrobial, and antitumor activities[3,4].

Currently, there are 12 main categories to group cucurbitacins and their derivatives according to their side-chain variations[4]. There are 17 main molecules from cucurbitacin A to cucurbitacin T, and hundreds of derivatives from them. Among those, cucurbitacin B, D, E, I, and their derivatives have been studied extensively for their strong anticancer activities[3,4]. Also, cucurbitacin F, O, P, Q, and their derivatives are known to have modest anticancer activities[4]. However, care should be taken in that not all cucurbitacin derivatives from the same category retain the same anticancer activity. For example, cucurbitacin D showed significant anticancer activity in many human cancer cell lines, whereas its 2-O-glucoside derivative did not[4].

Cucurbitacins drew the attention of drug industry in the 1960s when some early publications showed their anticancer activities to a variety of cancers *in vitro* and *in vivo*[3,4]. It is not clear why the initial attention to cucurbitacins withered for the following 2 decades. Some articles state that the loss of interest may be due to the low therapeutic index and nonspecific cytotoxicity of cucurbitacins[4]. Some human case reports on cucurbitacin poisoning could also be part of the reason[5,6]. However, many early studies showing cucurbitacin toxicity[3,4,5,6] used the plant extract directly without proper purification and failed to provide not only any clinically relevant information, but also standardized methods to study pharmacological and biological properties of cucurbitacins[1,2].

In the late 1980s, the development of the NCI60 human tumor cell line anticancer drug screen by the National Cancer Institute (NCI) gave a big boost to the research regarding plant-derived anticancer compounds, including cucurbitacins[1,7]. The NCI60 cell line screen provided a standardized tool to study cucurbitacins in a systematical way and many cucurbitacin studies started to reappear in the 1990s. Another notable change was that cucurbitacin researchers started to analyze the biological mechanism of action of cucurbitacins at the molecular level. Selected studies regarding antitumor activity of cucurbitacins since 1990 will be discussed in the following.

## RESPONSE OF CANCER CELLS TO CUCURBITACIN EXPOSURE

Like other plant-derived substances, cucurbitacins induce several morphological and physiological changes in cancer cells. Drastic changes in cell shape, such as rounding, swelling, pinocytic blebbing, submembranous inclusions, and blisters, are observed within a couple of hours. Some of the morphological changes could be explained by the dysregulation of cytoskeleton homeostasis by cucurbitacins. Duncan et al. reported the dramatic increase in F-actin to G-actin ratio and abnormal reorganization of the vimentin network by cucurbitacin E in human prostate cancer cell lines[9,10]. Studies with cucurbitacin B in our group also showed the aggregation of F-actin in various human cancer cell lines[11,12,13], which implies the disruption of the dissociation process of F-actin to G-actin by an unknown mechanism. However, unlike vinca alkaloids and taxanes, there is no clear evidence that cucurbitacin affects the microtubule network.

Multinucleation is another common morphological change that was consistently reported in human cancer cell cultures exposed to cucurbitacin for more than 24 h. According to Duncan et al., multinucleation implies that cucurbitacin blocks cytokinesis, but not karyokinesis[10]. This is in conjunction with the observation that actin (which is involved in cytokinesis) is disrupted, whereas microtubule (which is involved in karyokinesis) is not.

Multinucleation can result from the disruption of the cell cycle. Many reports showed that cucurbitacins induced cell cycle arrest, mostly in G2/M phase[13,14,15,20], but S-phase arrest in HL-60 and U937 human leukemia cell lines was also reported by our group[11]. G2/M arrest happens in the early period of cucurbitacin exposure and results in apoptotic death of the tumor cells[20]. Tannin-Spitz et al. showed that G2/M arrest occurred in breast cancer cell lines (MCF-7 and MDA-MB-231) exposed to cucurbitacin B/E glucosides by the inhibition of cyclin-dependent kinase (cdk) $p34^{CDC2}$ and cyclin B1, both in expression level and activation status[15]. Our group also showed G2/M arrest by up-regulation of cdk inhibitor $p21^{WAF1}$, and by down-regulation of cyclin A and cyclin E in pancreatic cancer cell lines (Panc-1 and MiaPaCa-2) exposed to cucurbitacin B[14].

## CUCURBITACINS AND THEIR MOLECULAR MECHANISM OF ACTION

By what molecular mechanism do cucurbitacins achieve the cell cycle arrest, apoptosis, and growth suppression of the cancer cells? There are several oncogenic signaling pathways that are commonly involved in cancer cell proliferation and survival. The JAK-STAT pathway, the Akt-PKB pathway, and the MAPK pathway are important pathways in cancer cells and are also targets of the cucurbitacin family.

The JAK-STAT pathway induces Janus-kinases (JAKs) and Signal Transducers and Activators of Transcription (STATs), and regulates cytokine and growth factor signals. In many cancer cells, constitutive activation of STAT3 and STAT5 has been known to play important roles in tumorigenesis[8]. After the initial finding by Blaskovich et al. that cucurbitacin I (JSI-124) is a dual inhibitor of STAT3 and JAK2[16], many studies confirmed that cucurbitacin I is a powerful JAK-STAT inhibitor by blocking the tyrosine phosphorylation of STAT3 and JAK2 in various human cancers[15,16,17,18,19,20]. However, cucurbitacin I did not affect other oncogenic signaling pathways, such as the Akt-PKB or MAPK/ERK pathways[16].

Furthermore, our group discovered that cucurbitacin B inhibited the tyrosine phosphorylation of STAT3, STAT5, and JAK2 in pancreatic cancer cell lines (Panc-1 and MiaPaCa-2) *in vitro* and in Panc-1 xenografts *in vivo*[14]. Inhibition of the JAK-STAT pathway affected various downstream targets involved in progrowth signaling (e.g., c-myc, cyclins, survivin) and apoptosis (e.g., p53, Bcl-xL, Bcl-2)[8,17]. Therefore, G2/M arrest and apoptosis in pancreatic cancer cells exposed to cucurbitacin B could be explained as a result of inhibition of the JAK-STAT pathway and were confirmed by down-regulation of $p21^{WAF1}$, cyclin A, and cyclin E, and up-regulation of Bcl-xL. Like cucurbitacin I, cucurbitacin B did not inhibit other progrowth signaling pathways, such as the Akt/PKB and MAPK/ERK pathways in pancreatic cancer cells[14]. Interestingly, Sun et al. found that cucurbitacin A, which only differs with cucurbitacin B by its C-11 hydroxyl group, lost its activity as a STAT3 inhibitor while maintaining its activity as a JAK2 inhibitor[17], showing that the inhibition of STAT3 and JAK2 may follow different molecular mechanisms.

The anticancer mechanism of cucurbitacin in breast cancer cells is still not clear. Tannin-Spitz et al. exposed breast cancer cell lines (MCF-7 and MDA-MB-231) to cucurbitacin B/E glucosides and found that cucurbitacins increased the tyrosine phosphorylation of STAT3, unlike other cancers[15]. Considering the activated JAK-STAT pathway in breast cancer[8], this result was contradictory. The authors hypothesized that concurrent inactivation of PKB in a cell type–specific manner might explain this unique regulation, which requires further research.

Considering the important role of STAT3 during inflammation[21], it is not surprising that part of the anticancer activity of cucurbitacins is linked to their anti-inflammatory activity. Chronic inflammation can make individuals predisposed to many types of cancer[21]. Cucurbitacins seem to affect both cancer cells and normal macrophages through different mechanisms. In cancer cells, cucurbitacins work as STAT3 inhibitors, and make cells more susceptible to the attack of reactive oxygen species (ROS) and free radicals during inflammation[24]. In normal macrophages, however, cucurbitacins work as inhibitors of the IKK/NF-κB pathway rather than the inhibitors of STAT3[23,24]. Inhibition of the IKK/NF-κB pathway by cucurbitacins results in the inhibition of key inflammatory enzymes, such as cyclooxygenase-2 (COX-2) and inducible nitric oxide synthase (iNOS), whose overproduction contributes to tumorigenesis[22,23,24]. However, it is still not clear how cucurbitacins can selectively choose their target pathway depending on the cell types.

Interestingly, when Escandell et al. used two human colon cancer cell lines that do not have activated STAT3 (HCT116 and Hke-3), 23,24-dihydrocucurbitacin B and cucurbitacin R still suppressed the tumor growth at significant level[25]. Furthermore, the presence of active kRas in HCT116 cells showed more protection from apoptosis than Hke-3 cells, which do not have active kRas[25]. Since kRas is upstream of ERK, the result implies the effect of cucurbitacins on the MAPK pathway.

Indeed, the MAPK signaling cascade was another target that cucurbitacins acted upon. Our group showed that cucurbitacin B affected the MAPK pathway in glioblastoma multiforme (GBM) cells *in vitro*[13]. Cucurtitacin B–treated GBM cells showed an increased level of phosphorylated p38, phosphorylated JNK, phosphorylated c-Jun, as well as a decreased level of phosphorylated ERK at the same time. Up-regulation of JNK may induce apoptosis in GBM cells, and down-regulation of p38 and ERK may block the cytokine signaling. Chan et al. also showed that both phosphotyrosine-STAT3 and phospho-ERK were suppressed in the K562 leukemia cell line[26]. Increased JNK phosphorylation by cucurbitacin D in the Hep3B human hepatocellular carcinoma cell line was also reported[27].

The Akt-PKB pathway mediates signals from receptor tyrosine kinases (RTKs) and integrins. Currently, no cucurbitacins are known to inhibit the phosphorylation of Akt. Although Tannin-Spitz et al. showed the down-regulation of PKB in breast cancer cell lines[15], further research is required to confirm the effect of cucurbitacin on the Akt-PKB pathway.

## SYNERGISTIC EFFECT OF CUCURBITACINS WITH CHEMOTHERAPEUTIC AGENTS

Despite its excellent anticancer activity, clinical use of cucurbitacin has challenges to overcome, such as low therapeutic index and nonspecific toxicity. One of the solutions to these problems can be the use of cucurbitacins in combination with known chemotherapeutic agents. In the clinic, many anticancer drugs are used together in combinations, not only to enhance the efficacy of the treatment, but also to avoid the buildup of resistance in cancer cells. Moreover, some drug combinations show strong synergism that helps to achieve the same therapeutic effect with lower dose and, hence, less toxicity. Encouragingly, some reports have shown that cucurbitacins show a synergistic effect with chemotherapeutic agents that are already established in the treatment of human cancers.

Sadzuka et al. showed that cucurbitacin E promotes cellular accumulation of doxorubicin, both by facilitating influx to and by preventing efflux from the tumor cells, implying synergistic effects of the two drugs[28]. Another study by Ramalhete et al. using cucurbitacin derivatives from *Momordica balsamina* confirmed statistically the synergistic effect of some cucurbitacin derivatives and doxorubicin using the fractional inhibitory concentration index (FIX)[29]. Recently, the synergistic effect of cucurbitacin B with gemcitabine in pancreatic cancer was discovered by our group[14]. Using an isobologram and combination index (CI) method, we showed that in a certain concentration range, cucurbitacin B and gemcitabine showed a CI value less than 0.9, which showed synergism between two drugs *in vitro*.

Strikingly, cucurbitacin B induced no apparent toxicity *in vivo*[14]. As a single agent at high dose (1.0 mg/kg), cucurbitacin B induced slight body weight loss after the first treatment, but no further toxicity was observed during the 7 weeks of treatment. We extensively searched for any signs of toxicity after treatment using various immunohistochemistry stainings on major organs such as liver, spleen, and kidney, as well as blood and serum chemistry tests. However, we found no signs of toxicity by cucurbitacin B. Considering the high dose of cucurbitacin B near $LD_{50}$ value (1.1 mg/kg)[5], the result was remarkable.

As Raikhlin-Eisenkraft et al. pointed out, many factors can affect the toxicity of cucurbitacins[6]. Bioreactivity of a compound can vary greatly depending on the presence of other compounds and the microenvironment *in vivo*. The discrepancy between our results and previous case reports can be partly explained by the extent of purity of cucurbitacins. Rapid advances in purification technology over the decades may have eliminated the other impurities coeluted with cucurbitacins from the plant source, which may be the true cause of toxicity in the past. Changes in the type of solvents for elution and dilution can also play a role. Defective immune systems in athymic nude mice can be another possibility. For this reason, we suggest in this review that cucurbitacin toxicity in humans needs to be restudied and should not be the reason to rule out cucurbitacin as a potential anticancer drug.

## CONCLUSION

Although neglected for decades, cucurbitacin is once again attracting attention as a potential anticancer drug. Its role as a JAK/STAT inhibitor, a MAPK modulator, and a cytoskeleton disruptor qualify it as an excellent candidate for clinical investigation. Cucurbitacin's synergism with already established chemotherapeutic agents is another big advantage. More studies both *in vitro* and *in vivo* on a variety of cancers will confirm the usefulness of cucurbitacin and reinvite this old drug to the modern clinic.

## ACKNOWLEDGMENTS

Grant support was received from Deutsche Forschungsgemeinschaft Grant TH 1438/1-1 for N.H. Thoennissen.
